# Electrokinetic Remediation of Cu- and Zn-Contaminated Soft Clay with Electrolytes Situated above Soil Surfaces

**DOI:** 10.3390/toxics12080563

**Published:** 2024-08-02

**Authors:** Zhaohua Sun, Jingxian Geng, Cheng Zhang, Qiu Du

**Affiliations:** 1 School of Transportation and Civil Engineering, Nantong University, Nantong 226019, China; 1814021013@stmail.ntu.edu.cn (J.G.); 18806290917@163.com (C.Z.); 15183097746@163.com (Q.D.); 2 Key Laboratory of New Technology for Construction of Cities in Mountain Area, Ministry of Education, Chongqing University, Chongqing 400045, China

**Keywords:** electrokinetic remediation, soft clay, heavy metal, citric acid, removal efficiency, electrolyte

## Abstract

Electrokinetic remediation (EKR) has shown great potential for the remediation of in situ contaminated soils. For heavy metal-contaminated soft clay with high moisture content and low permeability, an electrokinetic remediation method with electrolytes placed above the ground surface is used to avoid issues such as electrolyte leakage and secondary contamination that may arise from directly injecting electrolytes into the soil. In this context, using this novel experimental device, a set of citric acid (CA)-enhanced EKR tests were conducted to investigate the optimal design parameters for Cu- and Zn-contaminated soft clay. The average removal rates of heavy metals Cu and Zn in these tests were in the range of 27.9–85.5% and 63.9–83.5%, respectively. The results indicate that the Zn removal was efficient. This was determined by the migration intensity of the electro-osmotic flow, particularly the volume reduction of the anolyte. The main factors affecting the Cu removal efficiency in sequence were the effective electric potential of the contaminated soft clay and the electrolyte concentration. Designing experimental parameters based on these parameters will help remove Cu and Zn. Moreover, the shear strength of the contaminated soil was improved; however, the degree of improvement was limited. Low-concentration CA can effectively control the contact resistance between the anode and soil, the contact resistance between the cathode and soil, and the soil resistance by increasing the amount of electrolyte and the contact area between the electrolyte and soil.

## 1. Introduction

Soil heavy metal pollution refers to a phenomenon where the content of heavy metal elements, such as Cu and Zn, in the soil exceeds environmental quality standards or poses potential risks to ecosystems and human health. It is a crucial challenge in today’s environmental problems. Sources of Cu pollution include pesticide production, the chemical industry, mining, and metal processing, while Zn pollution includes plumbing, metal plating, brass manufacturers, and refineries [1]. In 2004, Cu and Zn were found to have a 30% and 70% contribution to the potential for ecotoxicity in Europe [2]. A recent survey study carried out to assess heavy metal contamination in farmland and urban soils showed that the maximum Cu contamination was recorded in Africa, followed by India, Iran, England, Wales, China, and America in decreasing order. Likewise, Zn contamination was also recorded as the highest in Africa, followed by India, Iran, China, and the Netherlands [3]. Heavy metals cannot be decomposed by soil microorganisms and are, therefore, prone to accumulation.

To solve this problem, many remediation techniques for cleaning heavy-metal contaminated soils have been developed, such as solidification stabilization [4,5], bioremediation [6,7,8], and leaching [9,10]. Electrochemical technology for the abatement of organic matter in water is also worth borrowing and referencing [11,12,13]. Compared with these technologies, electrokinetic remediation (EKR) is a promising technology due to its feasibility in the remediation of fine-grained soils contaminated by heavy metals [14,15,16]. The EKR technique, which is usually used as an in situ remediation system, can accelerate the mobility of charged contaminants by employing a low-level direct current across the contaminated soil [17,18]. However, there are some difficulties in in situ practice that limit the field application of the EKR technique [19]. One of the limitations regarding large-scale applications is the injection of anolyte and catholyte, which are prone to leakage, into the soil, thereby increasing the electrolyte dosage and causing secondary pollution to the soil [20]. Special precautions must be taken to control the occurrence of potential electrolyte leakage into surrounding soils. 

Considering the high moisture content and low permeability of soft clay with hydraulic permeability coefficients typically ranging from 10^−4^ to 10^−9^ cm/s [21], an electrokinetic remediation method using electrolytes placed above the ground surface is proposed. For example, in polluted soft clay with a hydraulic permeability coefficient of 5 × 10^−7^ cm/s, it would take approximately 6.3 years for the electrolyte to penetrate 1 m vertically through the soil under gravity. In view of these characteristics, an electrolyte chamber situated above the soil surface was designed and proposed for contaminated fine-grained soil by Sun et al. [22]. Thus, the removal efficiency in different soil sections was relatively uniform, and more than 90% of the initial Zn was removed. This method was also conducive to avoiding electrolyte solution leaks and alleviating secondary pollution.

The chemically integrated EKR technique, with the addition of a chemical agent (such as organic acid), has been confirmed to be an effective approach for changing the soil environment, improving the removal efficiency, and shortening the remediation time [14,23,24]. Citric acid (CA), when used as an electrolyte, has been found to be an enhanced chelating agent for the removal of many metals. Owing to its biodegradability, relatively low cost, and rather easy manageability, CA is considered a suitable chelating agent for in situ remediation. Moreover, the presence of increasing citrate concentrations in the interstitial fluid favors electro-osmotic flow [25,26]. The combination of soil acidification and enhanced electro-osmotic flow leads to excellent Cu and Zn removal, with removal rates higher than 70% [27,28]. Conversely, if the electro-osmotic flow appears at relatively low levels, the removal rate of heavy metals remains low even when high concentrations of CA electrolytes are used [24].

In general, the global efficiency of EKR depends on the combination of electrode processes, electromigration, diffusion, electro-osmotic flow, and Joule effect, together with the adsorption phenomena of contaminants on the matrix [29]. At present, different design parameters in laboratory and fieldwork, such as the concentration, dosage, and pH of the chemical agent, electrode optimization, heavy metal concentration, and voltage gradient, have been conducted to explore their influence on these effects and their contribution to EKR removal efficiency [30]. For Cu and Zn mixed pollutants, studies have found that Zn showed higher mobility in soil than Cu, and the pH required for Zn precipitation is higher than that required for Cu precipitation [31]. In reports, Cu was usually removed at lower rates than Zn [32,33,34]. However, the optimal design parameters for the EKR of Cu- and Zn-contaminated soft clay and its removal efficiency using the novel experimental method (electrolyte chamber situated above the soil surface) are not yet clear.

In this context, a set of EKR tests for Cu- and Zn-contaminated soft clay was performed using a novel experimental method (electrolyte chamber situated above the soil surface). CA at different concentrations and dosages enhanced these tests in terms of different heavy metal concentrations. The objective of this study is to evaluate and compare the heavy metal removal efficiency and changes in soil properties to provide guidance for the optimum selection of electrolytes to enhance the EKR efficiency. The results of this study are of great significance to the comprehensive understanding of the mechanisms of electrokinetic removal of metals from soils. 

## 2. Materials and Methods

### 2.1. Chemicals

Perchloric acid, hydrochloric acid, and sulfuric acid of reagent grade used to digest soil samples were provided by the Analysis and Testing Center of Nantong University. The remaining chemicals were purchased from Sinopharm Chemical Reagent Co., Ltd. (Shanghai, China) and were of analytical grade. Deionized distilled water was used in all experiments. 

### 2.2. Simulated Zn- and Cu-Contaminated Soft Clay

Due to the diversity, complexity, and heterogeneity of actual heavy metal-contaminated sites, it is difficult to analyze the impact of target variables on the EKR efficiency of heavy metal-contaminated soil. The migration patterns of pollutants in soil are also difficult to determine. Therefore, the Cu- and Zn-contaminated soft clay used in this study was artificially prepared by a dry method. Clay powder was obtained from a mining company in Jiangning District, Nanjing, China. The initial physical properties of the clay powder are summarized in Table 1. The initial Zn and Cu concentrations in the clay powder were 102 and 0 mg/kg. The clay powder was first weighed, and the volume of distilled water required to reach a water content of 50% was measured and poured into a container. The mass of zinc sulfate (ZnSO_4_·7H_2_O) and copper sulfate (CuSO_4_·5H_2_O) required for a concentration of about 500 or 1000 mg of Zn and Cu per kg of dry soil was then weighed and added to a water container, respectively. The soil specimen was prepared by thoroughly mixing the clay powder with the zinc and copper solution using a mechanical stirrer to ensure homogeneous dispersion of the metals. Subsequently, the contaminated soil specimen was sealed with plastic film and placed in a non-light environment for a week to allow for zinc and copper adsorption by soil solids to take place and to reach equilibrium. 

### 2.3. Experimental Setup

**Figure 1:**
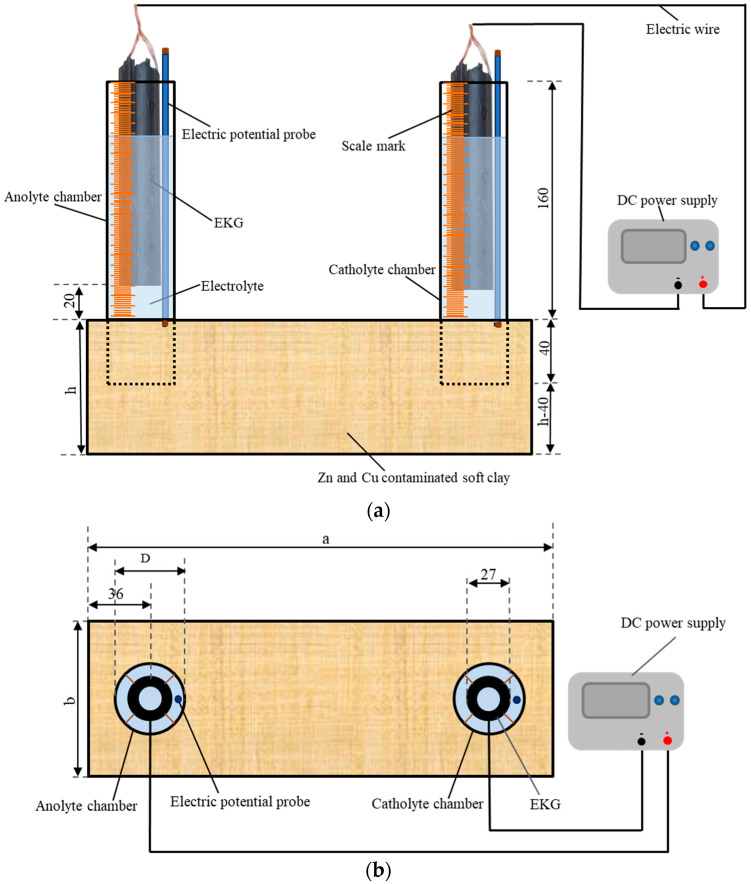

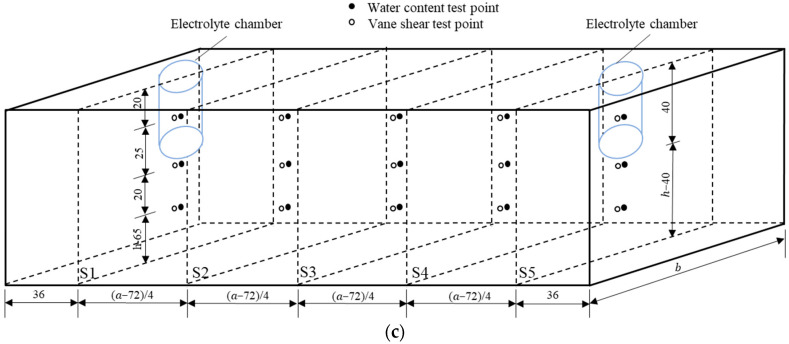
Schematic of the EKR experimental configuration: (**a**) front view, (**b**) top view, and (**c**) test point layout (unit mm).

The experimental device used was introduced by Sun et al. [22], and it is shown in Figure 1. Six rectangular plexiglass model boxes, specifically two with dimensions of 210 mm × 150 mm × 70 mm and four with dimensions of 300 mm × 100 mm × 90 mm (length a × width b × height h), were prepared. The electrolyte chamber (marked by scales on the outside) was an open-ended plexiglass tube. The volume variation of the electrolyte in the electrolyte chamber during the experimental period was monitored by measuring the scales. Six sets of tubes, each with two tubes, were used as electrolyte chambers. One set of the tubes had an outer diameter of 60 mm (D), a wall thickness of 2 mm, and a height of 200 mm, while the other five sets had an outer diameter of 45 mm (D), a wall thickness of 2 mm, and a height of 200 mm. After the artificially configured Zn- and Cu-polluted soft clay was loaded into the EKR cell for each test, a set of tubes was vertically pressed into the contaminated soil with a buried depth of 40 mm, as shown in Figure 1a,b. CA solution was obtained by dissolving citric acid monohydrate crystalline powder (C_6_H_8_O_7_ • H_2_O) in water. CA electrolytes with different concentrations and dosages were injected into the tubes (electrolyte chamber) according to the experimental schemes. Tubular electrokinetic geosynthetics (EKG) were chosen as the electrodes. The electrodes were described by Sun et al. [22]. It has been proved that the electrode has good conductivity and chemical stability and is basically noncorrosive. The EKGs were suspended in the electrolyte inside the electrolyte chamber with a bottom of electrode to soil surface distance of 20 mm. Conducting wires were used as connections between the direct current power supply (RXN-605D) and the EKGs.

### 2.4. Test Schemes

In essence, the EKR removal efficiencies of Cu and Zn were determined by the metal speciation in the soil matrix. The key to improving the EKR removal efficiency is improving the electromigration potential of the metal. The change in the electrolyte pH could significantly affect the migration behavior of metal ions. In this study, a CA solution was chosen as the anolyte and catholyte for the remediation of Cu- and Zn-contaminated soft clays with different levels of pollution. For mixed Cu and Zn pollutants, the feasibility of the novel experimental method (electrolyte chamber situated above the soil surface) needs to be confirmed and complemented with further analyses of the influence of the electrolyte concentration and dosage on the remediation efficiency. Table 2 illustrates that six EKR tests were designed for the experimental schemes. The dimensions of the model boxes of T1 and T2 were 210 mm × 150 mm × 70 mm, and that of T3–T6 were 300 mm × 100 mm × 90 mm. The electrolyte chambers of T2 tubes with an outer diameter of 60 mm were chosen, while other tests used electrolyte chambers with an outer diameter of 45 mm. The selection of two sizes of electrolyte chambers was intended to explore the impact of the contact area between the electrolyte and soil on the effectiveness of electrokinetic remediation. The specific size used for testing 0.2 M CA was chosen to explore whether increasing the contact area between the electrolyte and soil can enhance the electrokinetic remediation efficiency of low-concentration CA. This is because using a low-concentration CA solution in EKR offers benefits such as reduced cost, minimized environmental impact, and lower risk of corrosion.

The concentrations of Cu and Zn were 500 mg/kg in T1–T4 and 1000 mg/kg in T5–T6, respectively. To investigate the impact of CA concentration on the electrokinetic remediation efficiency, the anolyte and catholyte concentrations applied at T1 and T2 were both 0.2 mol/L, T3 and T5 were 0.5 mol/L, and T4 and T6 were 1.0 mol/L. In addition, electric potential probes were placed into the electrolyte chamber, in contact with the soil surface, to measure the effective electric potential of the polluted soil. The voltage was increased from 20 to 60 V in the experiment.

The DC power supply output voltage (U) that deducts the electrical potential drop at the anode–soil interface ($∆U_{as}$) and the electrical potential drop at the cathode–soil interface ($∆U_{cs}$) is called the effective electric potential ($U_{ef}$), and it has the following properties:

(1) $$U_{ef}={U-∆U}_{as}-{∆U}_{cs}$$

In the calculation process, the electrical potential drop at the anode–soil interface can be considered equivalent to the electric potential difference between the anode and the electric potential probe in the anolyte, while the electrical potential drop at the cathode-soil interface can be considered equivalent to the electric potential difference between the electric potential probe in the catholyte and the cathode. 

Due to the contact resistance between the electrode and the electrolyte, as well as between the electrolyte and soil, an electric potential drop was observed. Assuming that the contact resistance between the anode and the soil is $R_{as}$, the contact resistance between the cathode and the soil is $R_{cs}$, and the soil resistance is $R_{s}$, based on the current (I) in the circuit, it can be concluded that:

(2) $$R_{as}=\frac{{∆U}_{as}}{I}$$

(3) $$R_{cs}=\frac{{∆U}_{cs}}{I}$$

(4) $$R_{s}=\frac{U_{ef}}{I}$$

The daily working hours of each test were 12 h. Intermittent time was not included in the test results. During the experiment, the variations in current and electric potential in the soil were monitored using a multimeter every 2 h, and the volume changes of the anolyte and catholyte were monitored every hour. The electrolytes were refreshed one time per day. The replaced electrolyte was stored, and its pH was tested using a precision pH test paper. After each test, the shear strengths and moisture contents were tested according to the test point layout diagram shown in Figure 1c. These test points were distributed on five cross-sections (S1–S5) with different depths. The shear strength tests conforming to ASTM 2001 were conducted using a dynamoelectric vane shear (TT-LVS, manufactured by Zhejiang Geotechnical Instrument, Shaoxing, China). The vane shear apparatus had blades that were 25.4 mm in diameter, 25.4 mm in height, and 0.01 mm in thickness. The shear stress and rotation angle were automatically recorded. 

The soil sample was divided into three parts (anode area, middle area, and cathode area) by the boundaries of the S2 and S4 sections to obtain soil samples for heavy metal concentration testing. Each area was divided into nine equal grid volumes. In each grid, samples were taken in equal amounts to form a composite sample for testing the heavy metal content in the area. Mixed soil samples were collected at multiple points in equal amounts in each area and dried in an oven. Subsequently, the dried soil samples were ground and passed through a 0.15-mm sieve. Approximately 0.1 g of the powder soil sample was weighed and soaked in a mixed acid of HCl-HNO_3_-HClO_4_ for a night. The next day, the mixture was heated and deacidified using a heating plate with the temperature set to 200 °C in a fume hood. When the color of the mixture changed from ginger yellow to nearly light yellow and the soil sample changed from brown-yellow to gray-white, the mixture was removed from heat. To obtain the test solution, the digested soil sample mixture was diluted with deionized water to a volume of 40 mL and then filtered using a 0.22 μm needle filter. Finally, the heavy metal concentrations in the digestion solutions were measured in duplicate using an atomic absorption spectrophotometer (TAS-990F, manufactured by Beijing Puxi General Instrument Co., Ltd., Beijing, China). According to the heavy metal concentrations measured in the digested soil samples, the heavy metal removal rates (η) in the three parts of each test were calculated as follows:

(5) $$\eta =\frac{c_{0}-c}{c_{0}}\times 100\%$$

where *c*_0_ (mg/kg) is the initial heavy metal concentration in the polluted soil and *c* (mg/kg) is the heavy metal concentration in the soil after EKR treatment.

## 3. Results and Discussion

### 3.1. Electric Current

**Figure 2:**
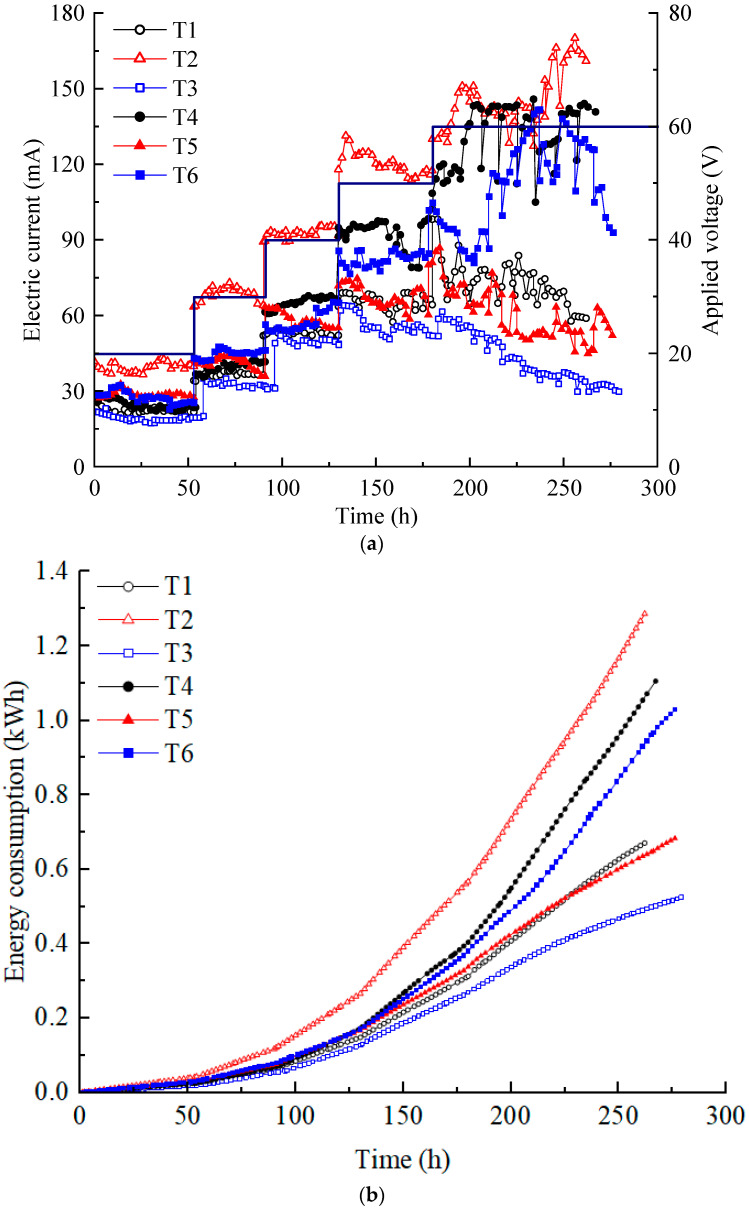
(**a**) Electric current variation; (**b**) energy consumption.

The current is a significant indicator of ion migration flux in soil systems. The magnitude of the current is affected by multiple factors, such as the content of H^+^ and OH^−^ produced by electrolysis of water molecules, the adsorption–desorption, complexation, precipitation–dissolution, and ion exchange of heavy metal ions in the contaminated soil [30]. Figure 2a illustrates the variation in the electric current in each test. The current increased in a step-like manner as the voltage was increased from 20 to 60 V, as applied by the DC power supply. The current at T2 was almost always the highest, while that at T3 was always the lowest. Although the electrolyte concentration of T2 was lower than T3–T6 and its heavy metal concentration was lower than that of T5 and T6, the current of T2 was almost always the highest. Because the T2 electrolyte chamber diameter of T2 was the largest, the contact area between the electrolyte and soil was the largest. Due to the influence of the dimensions of the model box, under the same voltage, the electric potential gradients of T1 and T2 were equal, and the electric potential gradients of T3–T6 were equal, with the former being higher than the latter. Moreover, T2 had the largest electrolyte volume; therefore, it contained more ions than T1 at the same electrolyte concentration. This indicates that the effect on the electric current of the contact area of the electrolyte and the soil, the electrolyte dosage, and the electric potential gradient is greater than that of the electrolyte concentration and the heavy metal concentration.

Compared with T1, although the concentration of electrolyte used in T3 increased, the electrode spacing (horizontal distance from the anode center to the cathode center) increased by approximately 90 mm, resulting in a lower electric potential gradient. Hence, the effect of the electric potential gradient on the electric current was greater than that of the electrolyte concentration. There was only a difference in electrolyte concentration between T3 and T4 and between T5 and T6. The higher electrolyte concentration had a higher current. When comparing T3 and T5 and T4 and T6, only the heavy metal concentrations differed. However, those with high heavy metal concentrations could not consistently sustain a high current. Under high voltage (50 V and 60 V), the increase in current at T4 and T6 is greater than the increase in current at T1, T3, and T5. Perhaps this result is because high voltage can stimulate the migration of ions better in high-concentration CA electrolytes. The phenomenon was more pronounced in T4 at low heavy metal concentrations. Based on the above analysis, it can be concluded that various factors affect the magnitude of the current. In order of importance, the contact area between the electrolyte and soil, electric potential gradient, electrolyte concentration, and heavy metal concentration.

In addition, the current showed a trend of a short rise and then a gradual decrease at all voltage levels during daily working hours. During the experiment, it was possible to determine the drainage situation of the soil based on the electrolyte volume change, and settlement on the soil surface was also observed. Decreases in soil moisture content and porosity lead to interference in the conduction path of the current [35]. In addition, if the heavy metals in the contaminated soil continuously migrate to the catholyte under the action of electro-osmosis and electromigration and finally are discharged with the replaced catholyte, the current will exhibit a downward trend [16]. A large contact area between electrolyte and soil, high voltage potential, and high electrolyte dosage or concentration used during EKR are often accompanied by dramatic chemical reactions such as electrolysis of water molecules and thermal effects. Although it causes a large current, a significant portion of the applied voltage is wasted on soil heating, which significantly affects the stability of the EKR system.

The energy consumption variations in each test are shown in Figure 2b. The total energy consumption of each test was small for the laboratory model test size. The energy consumption was the highest for T2, followed by T4 and T6. T1 and T5 had similar energy consumption, while T3 had the lowest energy consumption. In the proposed electrical scheme, variations in energy consumption are primarily influenced by changes in current. After 130 h, once the voltage reached 50 V, the energy consumption of each test increased almost linearly at a relatively high rate.

### 3.2. Electric Potential and Resistance

**Figure 3:**
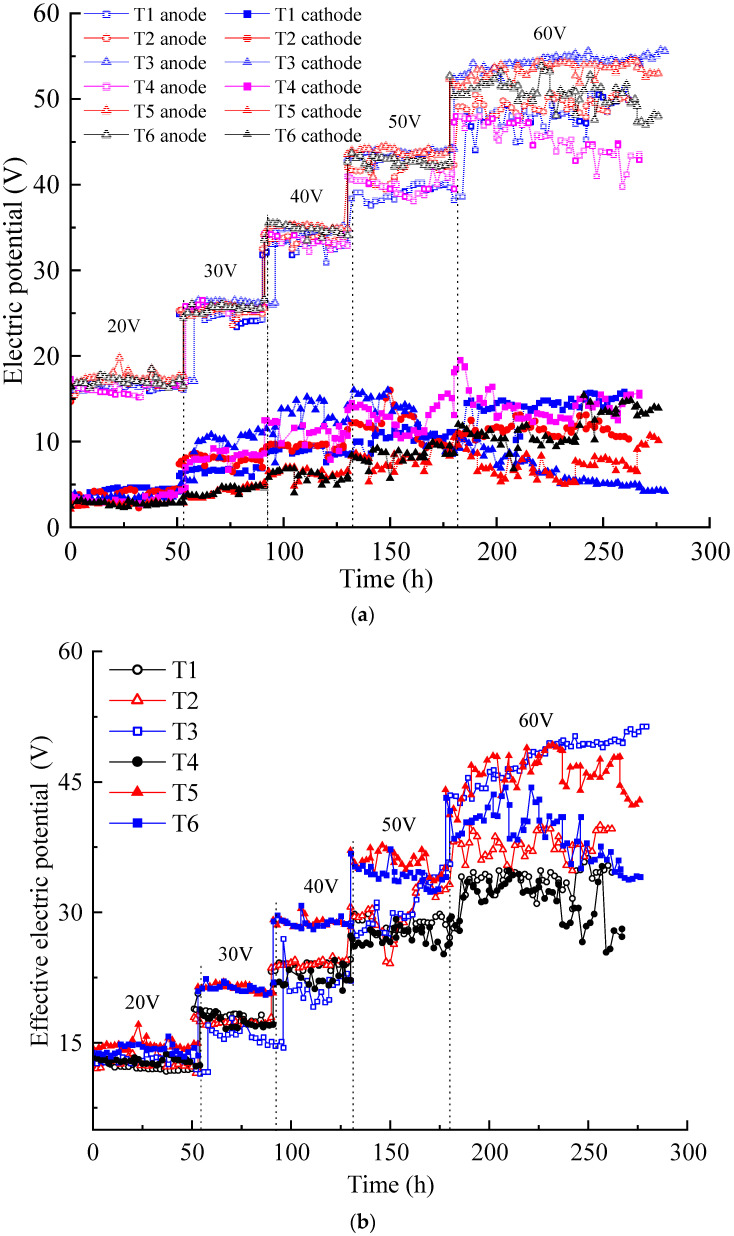
Electric potential variation: (**a**) electric potential of the soil near the anode and cathode; (**b**) effective electric potential of the contaminated soil.

Electric potential is the driving force for ion migration and electro-osmotic flow in contaminated soil pore fluids. As shown in Figure 1a, the electric potentials of the soil surfaces inside the anolyte and catholyte chambers were tested using the electric potential probes. The variation in the electric potential is shown in Figure 3a. Under the increased voltage applied by the DC power supply, the electric potential of the soil surface in the anolyte chamber was always higher than that in the catholyte chamber. The former trend showed a stepwise increase, while the latter trended relatively weak. The electric potentials of the soil surface in the anolyte chambers of the six tests were relatively close under applied voltages of 20, 30, and 40 V, and the difference increased under voltages of 50 and 60 V. The greater the applied voltage, the greater the electric potential loss of the soil surface in the anolyte chamber. These results indicate that the actual electric potential acting on the contaminated soil was lower than the applied DC power supply voltage.

Figure 3b illustrates the variation in the effective electric potential of each test during the EKR process. The effective electric potential in each test increased with the increasing of the applied voltage. Under the voltages of 20–50 V, the effective electric potential of T1–T4 was relatively close and lower than those of T5 and T6. When the voltage rose to 60 V, evident differences were observed in the effective electric potential in each test. The effective electric potential of T3 gradually surpassed those of other experiments and maintained a good upward trend, while T4–T6 showed a downward trend. An increase in the effective electric potential of T3 at an output voltage of 60 V may be due to electrode polarization.

**Figure 4:**
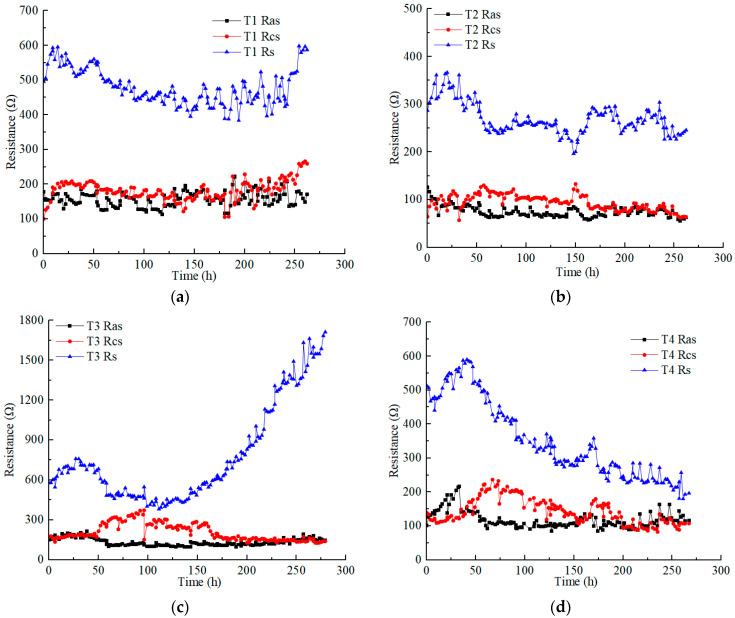

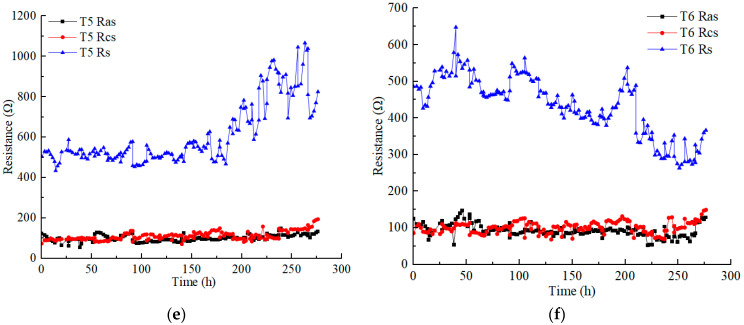
Resistance variation of anolyte, catholyte, and soil in each test: (**a**) T1; (**b**) T2; (**c**) T3; (**d**) T4; (**e**) T5; (**f**) T6.

Figure 4 illustrates the variations in the anolyte resistance ($R_{as}$), catholyte resistance ($R_{cs}$), and soil resistance ($R_{s}$) in each test under different applied voltages. Soil resistance is obviously higher than electrolyte resistance. The anolyte resistance was closer to or slightly lower than the catholyte resistance in each test. The anolyte resistance and catholyte resistance of each test ranged from 50 to 200 Ω, except the catholyte resistance of T3, which reached a higher value of nearly 400 Ω. The electrolyte resistance was affected by many factors, such as the contact area between the electrolyte and soil, the dosage and concentration of the electrolyte, and the conductivity of the contaminated soil (heavy metals concentration). However, in this experimental scheme, the aforementioned influencing factors constantly changed during the EKR treatment [30]. 

The anolyte concentration significantly affects soil resistance. Although the Cu and Zn concentrations of T5 were higher than those of T4, the soil resistance of T4 was lower than that of T5 at voltages above 30 V. High concentrations (1 M) of CA electrolyte reduced the difference in the original conductivity of contaminated soil. T3 and T5, using 0.5 M electrolyte, showed a significant increase in soil resistance at a voltage of 60 V. However, surprisingly, with the use of low-concentration electrolytes, the soil resistance of T2 remained almost stable.

On the whole, when using low CA concentrations, increasing the amount of electrolyte and the contact area between the electrolyte and soil can effectively control the anolyte, catholyte, and soil resistances. In addition, it is an effective measure to reduce costs. For example, during the daily working hours in these experiments, 220 mL of 0.2 M CA required a mass of 9.24 g C_6_H_8_O_7_ • H_2_O, while 120 mL of 0.5 M and 1 M CA required masses of 12.6 g and 25.2 g C_6_H_8_O_7_ • H_2_O, respectively.

### 3.3. Electrolytes pH

Pore water pH can affect the surface-charge properties of soil, metal speciation, and ligands of low-molecular acid with heavy metals. It has a significant impact on the desorption of heavy metals from the surface of soil particles into the liquid phase and subsequent migration. During EKR treatment, the anode surface undergoes electrolytic water–molecule reactions to generate oxygen and hydrogen ions, resulting in a decrease in the pH value. The surface of the cathode mainly undergoes Cu deposition, which reduces the amount of Cu ions that migrate to the catholyte. The chemical equations are as follows:

2H_2_O − 4e^−^ → O_2_ ↑ + 4H^+^ (anode) (6)

Cu^2+^ + 2e^−^ → Cu (cathode) (7)

The CA concentrations of the anolyte and catholyte in the six tests were 0.2 mol/L (T1 and T2), 0.5 mol/L (T3 and T5), and 1 mol/L (T4 and T6), with initial pH values of 2.5, 2, and 2, respectively. During the EKR treatment, the electrolytes were refreshed once per day (daily working hours were 12 h). The pH values of the replaced anolyte and catholyte were tested in time. The pH variations of the daily updated electrolytes in each test are shown in Table 3. After each test, the daily pH values of all replaced electrolytes were analyzed. The anolyte pH values of T1 and T2 basically dropped to about 2.0 each time, while their catholyte pH values remained unchanged at 2.5 under 20–50 V output voltage and increased to about 3.0 under 60 V output voltage. The anolyte pH values of T3–T6 were reduced to about 1.5, while their catholyte pH values were maintained at approximately 2.5. It can be seen that the catholyte pH value in each test increased by approximately 0.5, while the anolyte pH value decreased by approximately 0.5 after 12 h of use. The electrolytes were always kept in an acidic environment with low pH. Hence, the CA solution effectively controlled the pH of the anolyte and catholyte during the EKR treatment. This helps promote the transformation of heavy metals into free states in polluted soil and alleviates the precipitation of heavy metals [36,37].

### 3.4. Electro-Osmotic Flow

**Figure 5:**
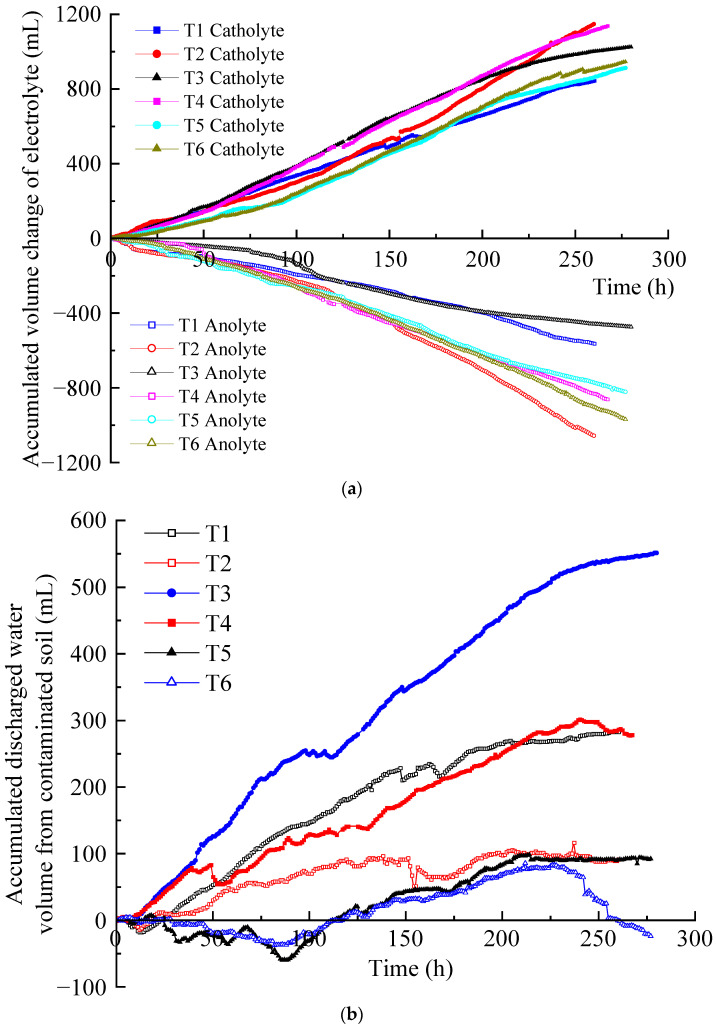
Electro-osmotic flow: (**a**) accumulated electrolyte volume change; (**b**) accumulated discharged water volume from the contaminated soil.

The migration of hydrated cations in the soil pore fluid to the cathode formed electro-osmotic flow under the action of an electric field. It is an important factor affecting the removal efficiency of EKR [27,28]. The accumulated volume changes of the anolyte and catholyte of T1–T6 are shown in Figure 5a. The difference between the accumulated volume increase in catholyte and the accumulated volume decrease in anolyte at the same time is the accumulated volume of discharged water from the contaminated soil, as shown in Figure 5b. It can be seen that in each test, the accumulated volume of the catholyte increased approximately linearly, whereas that of the anolyte decreased approximately linearly.

Through the accumulated volume change of the anolyte and catholyte in each test during the EKR process, it can be inferred that the volume reduction of the anolyte was positively correlated with the electric current. The larger the electric current, the greater the volume reduction of the anolyte. In this experimental system, the lowest decrease in accumulated anolyte volume usually leads to a higher water discharge volume in the soil. Based on these results, it can be inferred that at relatively low currents, the hydrated cations in the soil migrate to the cathode more easily, resulting in soil drainage. In contrast, a high current can drive more hydrated cations from the anode solution toward the cathode, which hinders the migration of the soil’s own hydrated cations and leads to poorer soil drainage. 

The increase in the volume of the catholyte and the decrease in the volume of the anolyte do not synchronously change. For example, for T1, during 0–5 h, the catholyte volume increase was slightly higher than the anolyte volume reduction, while during 6–21 h, the contrary was the case. Since then, the volume increment of the catholyte remained higher than the volume decrease of the anolyte, and the highest of the former was 1.86 times that of the latter. At the same time, the volume reduction of the anolyte in T2 was always higher than that in T1. The volume increment of the catholyte in T2 was higher than that in T1 before 32 h and slightly lower than T1 during 33–130 h. Thereafter, T2 was significantly higher than T1. The number of ions in the T2 anolyte was definitely higher than that in the T1 anolyte, indicating that after the voltage rose to 50 V, the electric current was further increased, which was more conducive to the migration of hydrated cations. In the end, the catholyte volume increment at T2 outperformed the other tests. T3, T5, and T6 also exhibited an alternating relationship between the increase in catholyte volume and the decrease in anolyte volume. Only T4 consistently maintained a larger increase in catholyte volume than a decrease in anolyte volume. In T1–T3 and T5, the phenomenon of a smaller increase in catholyte volume than a decrease in anolyte volume only occurred under low-voltage conditions. However, the increase in the catholyte volume was always lower than the decrease in the anolyte volume in T6 under both low and high voltage conditions. This resulted in the retention of electro-osmotic flow generated from the anolyte in the contaminated soil, exceeding the accumulated discharged water from the polluted soil.

In the EKR process, the electro-osmotic flow generated in the anolyte enters the contaminated soil, and possibly all or part of it migrates to the catholyte chamber along with that generated in the contaminated soil. As shown in Figure 5b, in each test, significant differences were observed in the accumulated discharged water volume of the contaminated soil. When the curve rises, the electro-osmotic flow generated in the contaminated soil is discharged into the catholyte chamber. When the curve is gentle, the polluted soil does not discharge water, and all the electro-osmotic flow generated in the anolyte is discharged to the catholyte chamber. The decrease in the curve indicates that some of the electro-osmotic flow generated in the anolyte is retained in the polluted soil. If the curve decreases but its value is not below zero, the result indicates that although the electro-osmotic flow generated in the anolyte is retained in the polluted soil, it does not exceed the accumulated discharged water of the polluted soil. If the curve drops below zero, the residual amount of electro-osmotic flow generated in the anolyte in the polluted soil exceeds the accumulated discharged water from the polluted soil.

The accumulated discharged water curves of the contaminated soil in each test displayed an upward, downward, or gentle trend. The final order of the accumulated water discharge volume of the contaminated soil was T3 > T1 ≈ T4 > T2 ≈ T5 > T6. The T2 curve did not rise much and had a gentle period for a long time, whereas the T5 and T6 curves had a relatively long period of decline, with some results below zero. Finally, only T6 had accumulated discharged water from the soil that was less than zero (−24 mL), indicating that 24 mL of anolyte remained in the contaminated soil at the end of the test. 

### 3.5. Water Content

**Figure 6:**
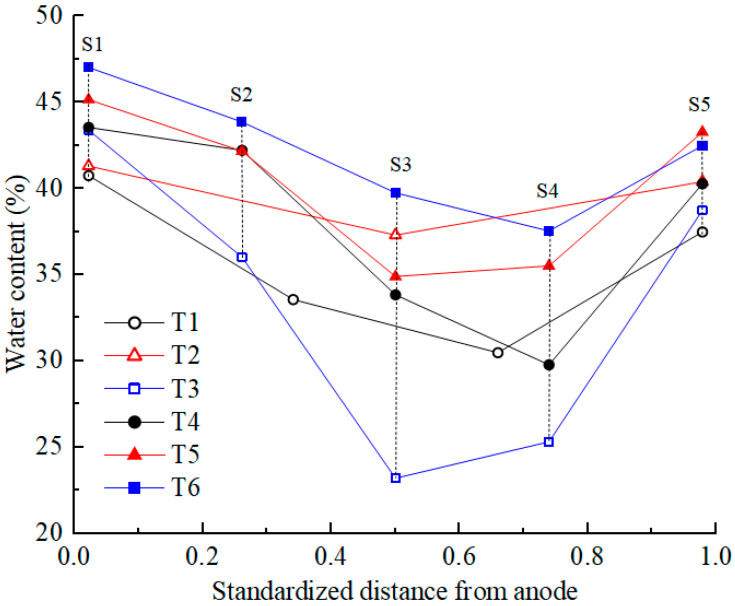
Water content distribution in the soil after EKR treatment.

After EKR treatment, soil water content was tested according to the testing point layout diagram shown in Figure 1c. The testing points were distributed at the surface, middle, and bottom positions of the five cross-sections (S1–S5). The average moisture content of each section was calculated, and its distribution is shown in Figure 6. Due to the development of cracks on the soil surface in T1 and T2, the measurement points deviated from their original positions. Compared with the initial moisture content, the moisture content of all soil sections after treatment decreased to varying degrees. For T4 and T6, the moisture content decreased from the S1 to S4 sections and increased at the S5 section. For the other four experiments, the moisture content decreased from the S1 to S3 sections and increased from the S4 to S5 sections. The position with the highest water discharge from the polluted soil was at the center between the two electrodes and leaned toward the cathode. It is mainly due to the fact that the pore water pressure of the soil in the middle of the test device is the smallest and that the electro-osmotic flow migrated toward the cathode. S1 and S5 were the cross-sections where the centers of the anolyte and catholyte chambers were located, respectively. Due to the direct contact between the soil surface of the S1 and S5 sections and the electrolyte, the moisture content of the soil was relatively high, with the former being higher than the latter.

The migration of electrolytes into the soil under gravity was negligible due to the low permeability coefficient of the contaminated soft clay. Under the action of a direct current electric field, the hydrated cations in the anolyte and the contaminated soil migrated toward the catholyte chamber. The accumulated discharged water from the contaminated soil was used to determine the moisture content. Based on the experimental results, the relationship of the average water content at all testing points was T3 < T1 < T4 < T2 < T5 < T6. The relationship between the accumulated discharged water from the contaminated soil was T3 > T1 > T4 > T2 > T5 > T6. The more water was discharged from the contaminated soil, the lower the moisture content [35]. Taking T3 as an example, under high voltages of 50 and 60 V, the accumulated volume of discharged water from the contaminated soil in T3 is significantly higher than that of the other tests. This leads to a relatively lower average soil moisture content in T3, as shown in Figure 6. Consequently, when compared to the other tests, the electrical resistance of the soil in T3 is relatively higher, and its current is notably lower.

In order to analyze the distribution of the electric field, which would be affected by the depth of the electrolyte chambers buried in the soil, the distribution of the water content in the depth direction was analyzed. In all experiments, it was found that the moisture content at the middle and bottom of the same section was relatively close. The moisture content of surface soil was lower than that of the middle and bottom soil due to evaporation (except for S1 and S5). In this study, the soil sample thickness was small; therefore, the electric field distribution along the depth direction does not significantly affect the electric field force of the hydrated cation migration in the contaminated soil.

### 3.6. Shear Strength

**Figure 7:**
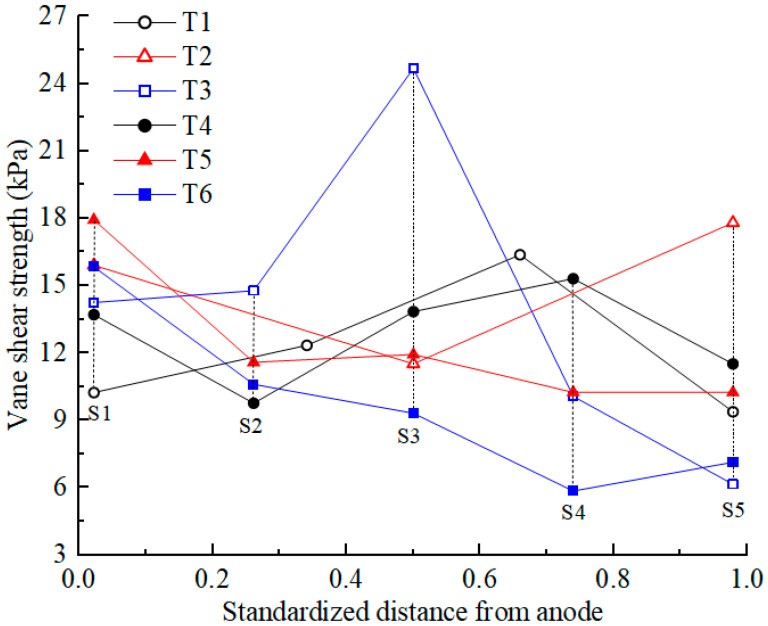
Vane shear strength of the soil after EKR treatment.

Shear strength is an important mechanical property of soil. After EKR treatment, the shear strength of the soil was first tested according to the testing point layout diagram shown in Figure 1c. The average shear strength at each section was calculated, and its distribution is shown in Figure 7. The initial shear strength of the contaminated soil was approximately 0. After EKR treatment, the shear strength of the soil varied from 6 to 26 kPa, indicating that EKR can improve the mechanical properties of contaminated soft clay, but the degree of improvement is limited. The factors that affected the shear strength of a certain section in each experiment were mainly related to drainage consolidation and chemical reinforcement at that location.

When the soil moisture content after EKR treatment decreased to below 25%, drainage consolidation was the dominant factor affecting the soil shear strength. The lower the moisture content was, the higher the shear strength became, such as in the S3 section of T3. When the soil moisture content after EKR treatment exceeded 25%, chemical reinforcement played a dominant role in the shear strength, and the influence of drainage consolidation was weakened. For example, the soil moisture content in the sections from S1 to S3 or S4 in each test decreased, whereas the average shear strength in these sections did not show an upward trend. In contrast, the S1 section, where the anolyte chamber was located, exhibited higher shear strength. This occurred because H^+^ in the anolyte entered the soil under the action of an electric field, resulting in silicate decomposition in the soil. The generated silicate ions reacted with calcium ions in the soil to produce hydrated calcium silicate (CSH), as demonstrated by the following reaction:

Ca^2+^ + SiO_3_^2−^ + H_2_O → CaSiO_3_ · H_2_O (8)

CSH is a binder that enhances the bonding effect of soil particles, thereby improving soil strength. Therefore, the closer the distance to the anode and the lower the pH of the soil, the more obvious the chemical reinforcement effect [38]. For example, in T3, the soil moisture content of the S2 section was 11% higher than that of the S4 section, but the shear strength of S2 was about 5 kPa higher than that of S4.

The soil near the cathode also has a chemical reinforcement effect, but this effect is not as obvious as that at the anode (except T2). During the daily working hours, the catholyte was always maintained in an acidic environment, but at night, after the electrolyte was removed, the surface of the soil inside the cathode chamber was exposed to air. Cations (Ca^2+^, Al^3+^, etc.) that migrate to the soil near the cathode react with carbon dioxide in the air to precipitate and promote the bonding of soil particles. Overall, the good electro-osmotic flow of T2 indicates that more cations migrate to the soil near the cathode, resulting in the best chemical bonding effect in the soil near the cathode.

### 3.7. Heavy Metal Concentration

**Figure 8:**
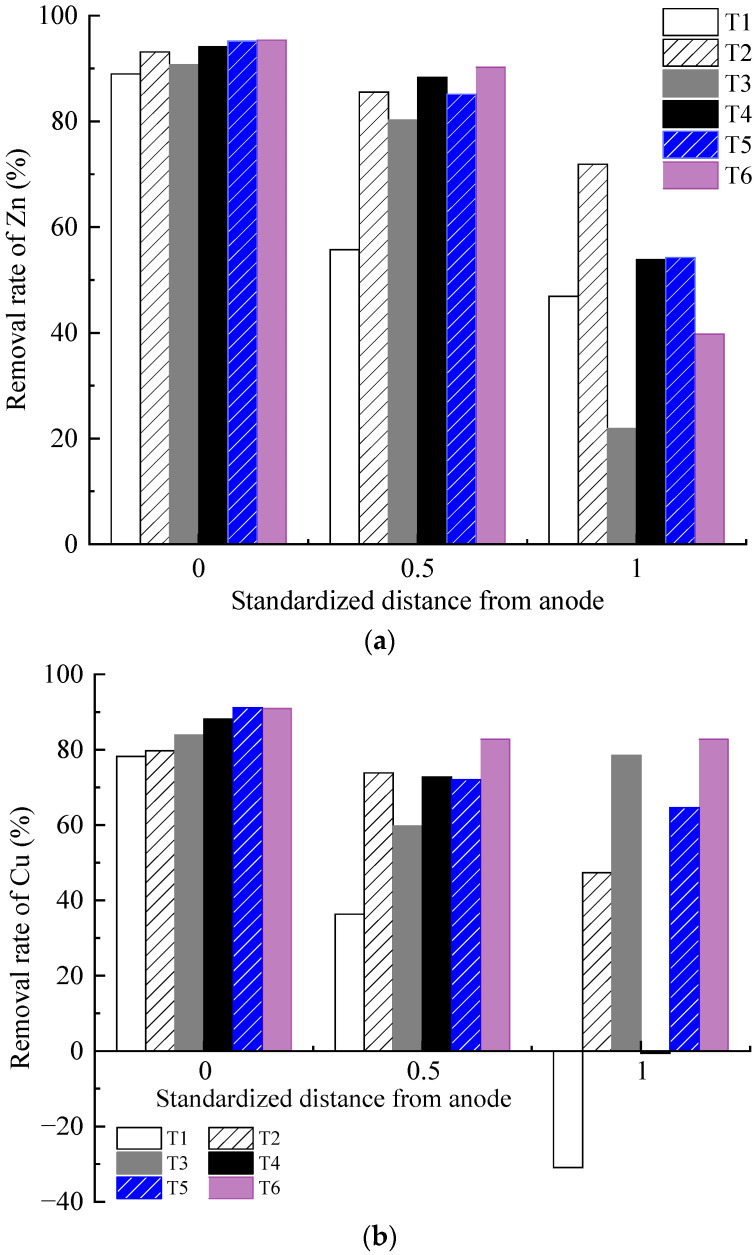

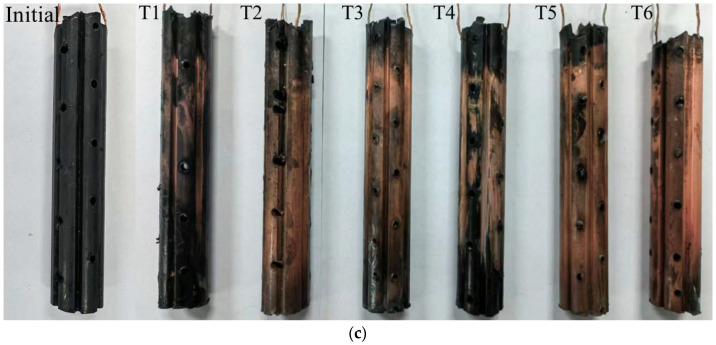
Heavy metal concentration distribution: (**a**) removal rate of Zn; (**b**) removal rate of Cu; (**c**) precipitation of Cu on the cathode surface.

The removal efficiency of each test is illustrated in Figure 8. The Zn and Cu removal rates of the contaminated soil decreased from anode to cathode areas. The heavy metal removal rate of the soil in the anode area was relatively close, whereas the difference in the heavy metal removal rate of the soil in the middle and cathode areas gradually increased. With the same heavy metal concentration, the removal rates of Cu and Zn in the soil in the anode region and the middle region increased with the increasing concentration and dosage of the CA electrolyte. For example, the Zn and Cu removal rates of T2 were higher than those of T1, T4 was higher than T3, and T6 was higher than T5. 

Under the action of an electric field, hydrogen ions in the anolyte (generated by the electrolysis of water molecules and contained within the CA electrolyte itself) migrate to the polluted soil. Thereafter, they compete with heavy metal cations for adsorption sites and exchange ions with heavy metal cations attached to the surface of colloidal soil particles, thereby promoting the desorption of heavy metal cations from negatively charged clay particles. After the heavy metal ions detach from the soil surface, they enter the liquid phase and migrate toward the cathode, or hydrated cations with polar water molecules migrate toward the cathode to form electro-osmotic flow. Moreover, the CA electrolyte can use its carboxylic acid and hydroxyl groups to chelate with Cu and Zn, forming stable chelates that are not easily adsorbed on soil. This reaction inhibits the adsorption of heavy metals on soil particles [10].

The results under different optimization parameters are compared in Table 4. The average removal rates of the heavy metal Zn in T1–T6 were 63.9%, 83.5%, 64.3%, 78.8%, 78.2%, and 75.2%, respectively. The average Zn removal rate in T2 was the highest. After comprehensive analysis, it was found that the main factor determining the removal rate of heavy metal Zn is the volume reduction of the anolyte. The volume reduction of the anolyte in T2 was the largest, while that in T1 and T3 was the smallest. The removal rates of the heavy metal Zn also exhibited the same magnitude correlation. The volume reduction of the anolyte represents the migration intensity of the electro-osmotic flow in the EKR system. A higher volume reduction of the anolyte is always associated with a higher Zn removal rate. The average removal rates of heavy metal Cu in T1–T6 were 27.9%, 67.0%, 74.0%, 53.4%, 75.9%, and 85.5%, respectively. The precipitation of Cu on the cathode surface after each test is shown in Figure 8c. The average Cu removal rate in T6 was the highest. After comprehensive analysis, it was found that the main factors affecting the Cu removal rate in sequence were the effective electric potential of the soil and the electrolyte concentration. The lower average Cu removal rates in T1 and T4 were due to Cu accumulation in the soil near the catholyte chamber. This is related to the relatively low effective electric potential of the soil in both experiments. Although the electrolyte concentration of T4 was 1.0 mol/L, it could not be further improved. When the applied voltage of the power supply was 20–50 V, there was not much difference in the effective electric potential of soil between T5 and T6. When the applied power supply voltage was 60 V, the effective electric potential of the soil in T5 was higher than that in T6. However, the high electrolyte concentration of T6 resulted in a higher Cu removal rate than T5. T3 maintained a high soil effective electric potential under a 60 V output voltage; thus, its Cu removal rate was ranked third, followed by T2.

Heavy metals in soil contaminated with high concentrations are usually more easily removed than those contaminated with low concentrations [36,39,40]. This occurs because adsorption sites with different binding energies are distributed on the surface of soil particles. When the pollution concentration of heavy metals is low, heavy metals first occupy the high binding energy sites on the soil surface, which belongs to specific adsorption and is difficult to desorb. When the heavy metal pollution concentration is high, heavy metals combine with low-binding-energy sites, reducing the average binding energy of soil and heavy metals, which belong to non-specific adsorption and are more prone to desorption. The removal of Zn was better than that of Cu. This result is a result of the stronger electronegativity and selective adsorption ability of Cu than of Zn under the action of an electric field. Therefore, compared to Zn, the soil has a stronger selective Cu adsorption capacity. Meanwhile, CA is tricarboxylic acid, Cu^2+^ and Zn^2+^ compete for adsorption and complexation sites during processing. Owing to the stronger adsorption affinity of Zn, CA preferentially chelates Zn. In terms of the total removal efficiency, Zn was superior to Cu under the same experimental conditions. Kirkelund et al. [32] also stated that the removal of Cu was dependent on the dominant organic fraction. In their report, 86% of Zn and 61% of Cu were removed from Haakonsvern sediments after 28 days of electrodialytic remediation. Compared to indoor electrokinetic remediation tests with an electrolyte chamber placed on both sides of the soil sample [31,37,41], through the reasonable design of experimental parameters, this experimental setup (with the electrolyte chamber positioned on the surface of the soil) achieved relatively higher efficiency in the electrokinetic remediation of clay contaminated with copper and zinc.

## 4. Conclusions

The experimental results of Cu and Zn removal from Cu/Zn-contaminated soft clay by the six EKR experiments are summarized as follows.

For Cu/Zn-contaminated soft clay with lower heavy metal concentrations (500 mg/kg), the following conclusions can be drawn. Low-concentration CA (0.2 M) can effectively increase the electric current and electro-osmotic flow and control soil resistance, thereby improving heavy metal removal efficiency by increasing the amount of electrolyte and the contact area between the electrolyte and soil. Under the same experimental conditions, a 0.5 M CA electrolyte outperforms the 1 M CA electrolyte in EKR. For Cu/Zn-contaminated soft clay with high heavy metal concentrations (1000 mg/kg), the electrokinetic remediation efficiencies of Cu and Zn using a 0.5 M CA electrolyte are balanced, with both values around 75%. Increasing the CA concentration to 1 M can further enhance the electrokinetic remediation efficiency of Cu.

Moreover, the average removal rates of the heavy metal Zn were in the range of 63.9–83.5% for these tests. The best performance was observed for the test with the largest volume reduction of the anolyte. The average removal rates of heavy metal Cu were in the range of 27.9–85.5% for these tests. The best performance was observed at the highest heavy metal and CA concentrations. After comprehensive analysis, it was found that the main factors affecting the Cu removal rate in sequence were the effective electric potential of the soil and the electrolyte concentration. After EKR treatment, the water content of the contaminated soft clay decreases to some extent, and the shear strength increases to some extent, indicating that EKR technology can improve the mechanical properties of contaminated soft clay, but the degree of improvement is very limited.

## Figures and Tables

**Table 1:** Characteristics of the clay powder used in this study.

Properties	Value
Particle size analysis	
Fine sand (%)	15
Silt (%)	49
Clay (%)	36
Water content (%)	6
pH	6.5
Liquid limit (%)	43
Plastic limit (%)	23
Permeability coefficient (cm/s)	4.2 × 10^−7^
Zn (mg/kg)	102
Cu (mg/kg)	0

**Table 2:** Experimental scenario for EKR tests.

Test Number	Initial Soil Moisture Content (%)	Initial Zn and Cu Concentrations (mg/kg)	Anolyte and Catholyte Concentrations (mol/L)	Initial Electrolyte Volume in Each Electrode Chamber Every Time (mL)	Total Processing Time (h) ^a^	Electrolyte Refresh Time	Applied Voltage (V)
T1	49.8	500	0.2 M CA	120	260	One time per day (total daily working hours were 12 h.)	20-30-40-50-60
T2	49.8	500	0.2 M CA	220	260		
T3	49.4	500	0.5 M CA	120	260		
T4	49.4	500	1.0 M CA	120	260		
T5	49.7	1000	0.5 M CA	120	260		
T6	49.7	1000	1.0 M CA	120	260		

^a^ The total processing time does not include the intermittent period.

**Table 3:** pH variation of the daily updated electrolyte in each test.

Test Number		T1–T2		T3–T6	
Electrolyte		Anolyte	Catholyte	Anolyte	Catholyte
Initial pH		2.5		2	
pH of the electrolyte after daily replacement	20 V	2	2.5	1.5	2.5
	30 V				
	40 V				
	50 V				
	60 V		3		

**Table 4:** Comparison of the EKR tests results under different experimental conditions.

Results Comparison		Test Number					
		T1 (500-0.2-120-138) ^a^	T2 (500-0.2-220-138)	T3 (500-0.5-120-228)	T4 (500-1-120-228)	T5 (1000-0.5-120-228)	T6 (1000-1-120-228)
Electric current variation range (mA)		20.6–98.2	38.4–130.0	19.7–61.7	27.2–79.0	32.7–75.3	32.6–95.8
Effective electric potential $U_{ef}$ variation range (V)		12.8–35.4	12.0–39.6	12.8–49.5	13.6–35.3	14.4–47.8	13.9–36.0
Soil resistance $R_{s}$ variation range (Ω)		383.8–598.6	196.6–366.7	381.7–1713.3	180.0–590.1	434.3–1067.0	363.6–647.8
Change in liquid volume (mL)	Anolyte ^b^	−560.7	−1065.0	−473.9	−858.2	−820.8	−968.9
	Soil ^c^	283.1	92.0	551.3	278.6	92.3	−23.9
	Catholyte ^b^	843.8	1148.0	1025.1	1136.7	913.1	945.0
Average water content (%)		35.6	39.7	33.3	37.9	40.2	42.1
Average shear strength (kPa)		12.1	15.1	14.0	12.8	12.4	9.8
Average heavy-metal removal rate (%)	Zn	63.9	83.5	64.3	78.8	78.2	75.2
	Cu	27.9	67.0	74.0	53.4	75.9	85.5

^a^ 500-0.2-120-138 represents the experimental heavy metal concentration—concentration of CA electrolyte—initial electrolyte volume-electrode spacing. ^b^ A negative sign indicates a decrease in electrolyte volume, whereas a positive sign indicates an increase in electrolyte volume. ^c^ A positive sign indicates that although the electro-osmotic flow generated in the anolyte is retained in the polluted soil, it does not exceed the accumulated discharged water from the polluted soil. Negative signs indicate that the residual amount of electro-osmotic flow generated in the anolyte in the polluted soil exceeded the accumulated discharged water from the polluted soil.

## Data Availability

Data will be made available on request.
